# Pseudohyperphosphatemia in a patient with relapsed multiple myeloma after bone marrow transplantation: A case report

**DOI:** 10.1002/ccr3.2264

**Published:** 2019-06-14

**Authors:** Kittrawee Kritmetapak, Sophon Dumrongsukit, Jittirat Jinchai, Panibud Wongprommek

**Affiliations:** ^1^ Division of Nephrology, Faculty of Medicine Khon Kaen University Khon Kaen Thailand; ^2^ Department of Medicine, Faculty of Medicine Khon Kaen University Khon Kaen Thailand

**Keywords:** multiple myeloma, paraprotein, phosphate, pseudohyperphosphatemia

## Abstract

Pseudohyperphosphatemia is a laboratory artifact characterized by falsely elevated serum phosphate mostly due to paraprotein interference on the conventional automated analyzer. Clinician recognition of this phenomenon and pre‐analytical preparation, including dilution or protein precipitation, can obviate unnecessary therapy and potentially unveil the diagnosis of paraproteinemia especially related to multiple myeloma.

## 1 INTRODUCTION

Spurious electrolyte disorders denote an artifactually increased or decreased serum electrolyte value that does not correlate with their existing serum levels. Patients with these disorders typically do not manifest clinical symptoms or signs of a particular electrolyte disturbance, and no specific treatment is required. Spurious electrolyte disorders encountered in clinical practice can occur in many patients; however, they are common in patients with hematologic malignancy associated with paraproteinemia, especially multiple myeloma.^1^ Herein, the authors report a case of pseudohyperphosphatemia (spurious hyperphosphatemia) in a patient with relapsed multiple myeloma after autologous hematopoietic cell transplantation and provide a concise review of its clinical implication.

## 2 CASE REPORT

A 55‐year‐old male patient presented to our hospital with fatigue and dizziness for 3 days. His past medical history was significant for multiple myeloma diagnosed 6 years earlier. He has undergone autologous stem cell transplantation 4 years ago followed by lenalidomide maintenance and remained in stable complete remission. During the follow‐up, the patient felt well until 3 months ago when he was incidentally discovered to have a new onset of hypercalcemia, hyperglobulinemia, and 80% plasma cells in bone marrow aspiration. Thus, he was diagnosed with relapsed multiple myeloma and he received three cycles of daratumumab‐based therapy. He had no history of herbal medicine use or laxative abuse. His current medications were folic acid, vitamin B complex, and low‐dose acyclovir prophylaxis. On physical examination, he was alert and oriented with markedly pale conjunctiva and a mild tenderness over thoracolumbar spines. The rest of the physical and neurological examination was unremarkable. Laboratory results are summarized in Table 1.

**Table 1:** Patient's laboratory findings

Parameters	Patient values	Reference ranges
Hemoglobin (g/dL)	5.5	13‐17
Mean corpuscular volume (fL)	82	80‐100
Leukocyte count (/mm^3^)	2630	4000‐10 000
Platelet count (/mm^3^)	93 000	150 000‐400 000
Blood urea nitrogen (mg/dL)	18.4	7‐20
Creatinine (mg/dL)	0.80	0.6‐1.2
Sodium (mEq/L)	136	135‐145
Potassium (mEq/L)	4.3	3.5‐5.0
Bicarbonate (mEq/L)	20	22‐26
Chloride (mEq/L)	100	95‐105
Calcium (mg/dL)	10.9	8.5‐10.2
Phosphorus (mg/dL)	17.6	2.5‐4.5
Magnesium (mg/dL)	2.4	1.6‐2.4
Total cholesterol (mg/dL)	119	<200
Total protein (g/dL)	11.2	6.7‐8.2
Albumin (g/dL)	2.9	3.5‐5.0
Globulin (g/dL)	8.3	2.0‐3.5
Total bilirubin (mg/dL)	0.3	0.1‐1.2
Direct bilirubin (mg/dL)	0.1	0.1‐0.3
Alanine transaminase (U/L)	12	7‐56
Aspartate transaminase (U/L)	19	10‐40
Alkaline phosphatase (U/L)	40	40‐140
25‐hydroxyvitamin D (ng/mL)	25.8	>30

Peripheral blood smear showed normochromic normocytic red blood cells, decreased platelets, and marked rouleaux formation but no hemolytic blood picture. The cause of pancytopenia in this patient is likely ascribed to bone marrow involvement with plasma cells. The serum protein electrophoresis revealed an obvious monoclonal spike in the gamma band, and a monoclonal IgG kappa protein was detected on serum immunofixation. Multiple osteolytic lesions, especially in the thoracolumbar spines, were also significantly more evident on the plain radiograph when compared to the previous result.

Nephrology team was promptly consulted for thorough evaluation of severe hyperphosphatemia and contemplating the potential role of hemodialysis. Considering that the clinical manifestations of hyperphosphatemia (eg, seizures and tetany) were absent, despite extremely elevated serum phosphate, our patient also had underlying multiple myeloma with hyperglobulinemia that was a risk factor of analytical interference; hence, additional investigations to confirm pseudohyperphosphatemia were performed. Serum phosphate levels had been measured repeatedly by the conventional phosphomolybdate ultraviolet (UV) assays (ammonium molybdate method) using the cobas 8000 analyzer (Roche Diagnostics Corporation), and the concentration results ranged between 16 and 24 mg/dL. In order to decrease the serum paraprotein concentrations, the original serum sample was diluted and measured again. Moreover, we took another blood sample from our patient and the sample was treated with 20% sulfosalicylic acid to remove the paraproteins prior to phosphate analysis with automated analyzer. After the pre‐analytical sample dilution or precipitation of serum paraproteins with 20% sulfosalicylic acid, the serum phosphate concentrations returned to the reference ranges (2.5‐4.5 mg/dL), indicating that the falsely elevated serum phosphate levels were ascribed to the biochemical interference, which confirmed the diagnosis of pseudohyperphosphatemia.

## 3 DISCUSSION

Phosphate disorder in patients with multiple myeloma is often related to light chain‐induced proximal tubular dysfunction resulting in renal phosphate wasting and hypophosphatemia.^2^, ^3^, ^4^ Nevertheless, hyperphosphatemia is less common unless the severe renal failure is present. Pseudohyperphosphatemia is a laboratory artifact characterized by falsely elevated serum phosphate in the absence of severely impaired renal function and clinical manifestations of hyperphosphatemia.^1^ Clinically discordant biochemical results together with the predisposing factor in the patient alert our team to be aware of the possibility of spurious hyperphosphatemia caused by paraproteinemia interference. To the best of our knowledge, this is the first case report describing pseudohyperphosphatemia in a patient with multiple myeloma after bone marrow transplantation.

The most common cause of hyperphosphatemia is reduced urinary phosphate excretion due to renal impairment particularly when estimated glomerular filtration rate was below 30 mL/min/1.73 m^2^. The other main causes of hyperphosphatemia include enhanced cell lysis (eg, tumor lysis syndrome, rhabdomyolysis), hypoparathyroidism, pseudohypoparathyroidism, and excessive vitamin D ingestion.^5^, ^6^ Although seen particularly in patients with decreased urinary phosphate excretion, hyperphosphatemia can also be caused by excessive phosphate administration especially phosphate‐containing laxative.^7^, ^8^, ^9^

Most hyperphosphatemic patients are usually asymptomatic though the clinical manifestations depend on the severity and onset of hyperphosphatemia. Patients with acute hyperphosphatemia typically present with signs and symptoms of hypocalcemia, such as perioral numbness, tetany, and seizure, whereas chronic hyperphosphatemia generally lead to the deposition of phosphate and calcium in soft tissues, resulting in vascular calcification and tumoral calcinosis in patients with chronic kidney disease.^10^, ^11^, ^12^ Hyperphosphatemia also blocks the conversion of 25‐hydroxyvitamin D to calcitriol, leading to concomitant hypocalcemia and the stimulation of parathyroid hormone secretion.

Our patients had neither apparent cause nor clinical characteristics of hyperphosphatemia, and serum calcium levels were also not decreased as would have been expected with the observed serum phosphate levels. Moreover, renal function was preserved, leading us to the interpretation that the measured serum phosphate levels were falsely elevated due to paraprotein interference. Phosphomolybdate UV assay is the most commonly used test for the determination of serum phosphate concentrations.^13^, ^14^ Serum inorganic phosphate reacts with an ammonium molybdate reagent in the presence of sulfuric acid to form an ammonium phosphomolybdate complex, of which UV absorbance is measured at 340 nm. Precipitation of monoclonal immunoglobulin in an acidic reagent increases serum turbidity and optical density, leading to enhanced UV absorbance, and eventually causes the falsely high serum phosphate levels.^15^ Both the concentration and physicochemical characteristics of the monoclonal immunoglobulin may determine the severity of spuriously increased serum phosphate levels since the addition of normal globulin into the phosphomolybdate UV assay has no effect on serum phosphate results.^16^ Most patients with pseudohyperphosphatemia were associated with IgG kappa monoclonal gammopathy possibly because IgG has a lower carbohydrate content compared with other immunoglobulins, so it can produce larger amounts of protein precipitation.^17^, ^18^ Pre‐analytical serum dilution, ultrafiltration of paraproteins, or acidic deproteinization prior to a measurement of serum phosphate can eliminate this spurious result.^13^, ^19^

Pseudohyperphosphatemia has been described in patients with paraproteinemia as multiple myeloma, in particular, Waldenström macroglobulinemia, and monoclonal gammopathy.^1^, ^14^, ^20^ Other metabolic abnormalities commonly associated with multiple myeloma include osteolytic hypercalcemia, pseudohyponatremia due to hyperglobulinemia,^21^, ^22^ hyperuricemia due to increased cell turnover, low serum anion gap due to increased cationic IgG paraproteins,^23^, ^24^ and Fanconi syndrome induced by nephrotoxic light chains. Our patient manifested not only pseudohyperphosphatemia but also moderate hypercalcemia caused by extensive osteolytic bone resorption. Vitamin D intoxication is a common cause of concurrent hypercalcemia and hyperphosphatemia; nonetheless, slightly low serum 25‐hydroxyvitamin D levels in our patient excluded the diagnosis.

Pseudohyperphosphatemia has also been reported in serum undergone prolonged refrigeration, the contamination of heparin or recombinant tissue plasminogen activator in the blood sample,^25^, ^26^ treatment with liposomal amphotericin B,^27^, ^28^ in vitro hemolysis with phosphate release from red blood cells, and the presence of hyperbilirubinemia or hyperlipidemia^29^; however, none of these factors presented in our patient.

The clinical implications of pseudohyperphosphatemia are explicit enough but depend essentially on a clinical recognition that the results are artifactual. Inappropriate treatment with oral phosphate binder can lead to true hypophosphatemia and adverse effects, such as muscle weakness, metabolic encephalopathy, and even heart failure.^30^ Consequently, persistent unexplained hyperphosphatemia in an asymptomatic patient should prompt the clinicians to search for paraprotein‐related diseases prior to injudicious use of phosphate‐lowering therapy.

## 4 CONCLUSIONS

In conclusion, pseudohyperphosphatemia should be recognized in severely hyperglobulinemic patients with unexplained hyperphosphatemia as well as relatively normal serum calcium and renal function. When pseudohyperphosphatemia is suspected, careful measurement of serum phosphate after sample predilution or deproteinization with sulfosalicylic acid will prevent unnecessary and potentially harmful therapeutic interventions.

## CONFLICT OF INTEREST

None declared.

## AUTHOR CONTRIBUTION

KK: contributed to treatment planning, and the preparation, review, and submission of the manuscript. DS, JJ, and WP: contributed to the preparation of patient profiles and reviewed the manuscript.
